# Co-designing the FOotpaths foR Adolescent MAternal Mental HeAlth (FOR MAMA) intervention for pregnant teens in Malawi

**DOI:** 10.1093/inthealth/ihaf007

**Published:** 2025-02-06

**Authors:** Wezi Mhango, Daniel Michelson, Darya Gaysina

**Affiliations:** School of Psychology, Pevensey 1 Building, University of Sussex, Falmer BN1 9QH, UK; Department of Psychology and Medical Humanities, P.O. Box 280, Zomba, University of Malawi, Malawi; School of Psychology, Pevensey 1 Building, University of Sussex, Falmer BN1 9QH, UK; Department of Child and Adolescent Psychiatry, Institute of Psychiatry, Psychology & Neuroscience, IoPPN, King's College London, 16 De Crespigny Park, London, SE5 8AB, UK; School of Psychology, Pevensey 1 Building, University of Sussex, Falmer BN1 9QH, UK

**Keywords:** adolescents, co-design, intervention, LMICs, mental health, perinatal

## Abstract

**Background:**

This study aimed to gain insights into stakeholders’ priorities and preferences for a scalable intervention for common mental problems among perinatal adolescents in Malawi.

**Methods:**

Participatory stakeholder workshops (n=9) were conducted iteratively according to the principles of the Person-Based Approach. Three stakeholder groups were recruited from one urban and one rural primary health centre in Zomba district, Malawi: perinatal adolescents (n=10), their family members (n=8) and healthcare workers (n=10). Framework analysis was conducted using intervention descriptors from the Template for Intervention Description and Replication checklist.

**Results:**

Participants emphasized the need for information on causes and symptoms of common mental problems and for developing coping strategies: a) those focused on external stressors—problem-solving, financial literacy and interpersonal skills—and b) emotion-focused approach behaviours—behavioural activation, relaxation and anger management. There was a strong preference for healthcare workers as intervention providers. Participants agreed on a brief antenatal intervention delivered weekly using both group and individual formats. There were positive views on both self-help and guided formats. All stakeholder groups felt there was a need for follow-up to ensure that adolescents correctly engaged with the intervention material.

**Conclusions:**

Findings informed the design of a brief multicomponent guided intervention for adolescents in the antenatal period.

## Introduction

Globally, 14% of adolescents give birth annually.^1^ Higher prevalences have been reported in low- and middle-income countries (LMICs),^2^ with Malawi having one of the highest adolescent birth rates (29%).^3,4^

Pregnant adolescents may experience school dropout, low social support, stigma, birth complications, pre-term birth and financial difficulties.^5–7^ These factors pose cumulative risks for perinatal mental health problems, especially in LMICs, where they are compounded by the lack of mental health services, lack of awareness of symptoms and personal beliefs about causes of mental illness.^8^ Systematic reviews of studies from LMICs have revealed pooled prevalence estimates of up to 34% in the antenatal period and 25.8% in the postpartum period.^9,10^ In Malawi, the overall pooled prevalence for perinatal depression is estimated at 18.9%,^11^ with a much higher prevalence (43.5%) among adolescents.^12^ However, perinatal mental health intervention studies focused specifically on adolescents are relatively scarce, especially in LMICs.^13–15^

Meaningful stakeholder involvement can help researchers identify the priorities of targeted stakeholders, thereby increasing the likelihood of developing an intervention that will be contextually appropriate and achieve positive outcomes.^16^ Moreover, successful intervention delivery requires an understanding of the stakeholders’ conceptualization of mental health, motivations and contexts in order to increase feasibility and acceptability.^17^

The present study aimed to co-design a mental health intervention for perinatal adolescents in Malawi—FOotpaths foR Adolescent MAternal Mental HeAlth (FOR MAMA)—using the Person-Based Approach (PBA). PBA seeks to guide intervention development by gaining a deep understanding of the psychosocial context and the perspectives of key stakeholders using iterative research.^18^ At the planning stage (stage 1) we conducted extensive formative research to ensure that context-specific evidence could be effectively integrated with relevant theoretical and empirical literature.^15,19^ The aims and key findings of stage 1 are summarized in Table 1.

**Table 1.** Formative data sources and key findings

Source/purpose	Sample	Key findings
Literature review: the review aimed to explore interventions for perinatal depression and anxiety	Systematic reviews (n=18)	Psychoeducational interventions were the most commonly delivered among adolescents Task-sharing approaches have been widely utilized, especially in LMICs Although some reviews reported on findings from LMICs, studies from African countries were lacking
Mixed methods systematic review and lived experience synthesis: this review sought to find out for whom, in what contexts and how psychoeducation works for the prevention and/or treatment of perinatal depression and anxiety in youth	Quantitative studies (n=8) Qualitative studies (n=12) Young Advisory Group (n=12)	Psychoeducation was included in effective multicomponent interventions for both depression and anxiety Psychoeducation can impact depression and anxiety by stimulating help-seeking and developing skills for self-management of stressors and symptoms Psychoeducation was deemed to be more engaging when delivered in visually appealing formats, when incorporating stories and when facilitated by a supportive, kind and non-judgmental provider Task-sharing (interventions delivered by nurses/midwives) was utilized in most of the effective interventions
Local stakeholder interviews to explore the contextual risk and protective factors for depression and anxiety among perinatal adolescents as well as the demand and supply-side barriers to addressing perinatal depression and anxiety among adolescents in Malawi and explore how the participants understood and responded to common mental health problems, including the terminology used to describe mental health problems. This facilitated a contextually relevant approach to co-designing a mental health intervention for adolescents in Malawi	Perinatal adolescents (n=14) Family members (n=4) Healthcare workers (n=8)	Diminished social support was seen as a risk factor while positive social support was identified as a protective factor Other risk factors for perinatal depression and anxiety included body and birth worries, role transitions, stigma towards teen pregnancy, school dropout and financial struggles Demand supply barriers to mental care included adolescents not being aware of the symptoms of depression and anxiety; adolescents attributing symptoms other causes such as witchcraft; adolescents mostly utilizing informal coping mechanisms such as self-isolation, sleeping and ignoring symptoms with the hope that they will disappear on their own Supply-side barriers to mental care included lack of trained personnel and private spaces where mental health services could be provided Most participants used the term *matenda a nkhawa* (literally translating to ‘illness of anxiety’ to refer to symptoms of both depression and anxiety without differentiating any further

The current study focuses on stages 2 (design) and 3 (development) of the PBA. Specifically, we sought to address the following questions, using participatory stakeholder workshops:

1. What intervention components might work best to address common mental problems in perinatal adolescents in Malawi?
2. Who are the potential non-specialists that could credibly deliver the intervention?
3. How can the intervention be efficiently delivered to perinatal adolescents in Malawi?
4. Where will the intervention be delivered to ensure practicability for both adolescents and intervention providers?

## Methods

### Study design and setting

Co-design workshops (n=9) informed by PBA^18^ were conducted from July to September 2022 in two primary health centres of Zomba district in Malawi, representing urban and rural areas respectively. Zomba district has one of Malawi's highest adolescent birth rates (34.9%).^20^ Ethical approvals were obtained from the Sciences & Technology Cross-Schools Research Ethics Committee at the University of Sussex (ER/WM90/2) and the University of Malawi Research Ethics Committee (protocol no. P.11/21/103).

### Participants

We recruited adolescents in the perinatal period (i.e. pregnancy and 1-y postpartum), family members living with the participating adolescents and healthcare workers who provide antenatal and postnatal services. Twelve participants (five perinatal adolescents, two family members and five healthcare workers) were recruited by contacting those who had participated in a previous study^19^ and had provided consent for further contact. In addition, five adolescents were recruited through antenatal clinics. In the first instance, a researcher (WM) provided basic information about the study during the antenatal clinics. Interested participants approached WM, who provided further details about the study both verbally and through a written information sheet. To achieve a representative sample and reduce bias, consecutive sampling was used, whereby all eligible participants were recruited sequentially until the predetermined sample size was reached.^21^ These newly recruited adolescents invited six family members who contacted WM and received information about the study. Five healthcare workers were identified through snowball sampling based on nominated contacts from other health workers who had participated in a previous study.^19^ The final sample comprised 10 perinatal adolescents, 8 family members and 10 healthcare workers (see Table 2).

**Table 2.** Participant characteristics

Participants (n)	Age range (years)	Female:male, n
Perinatal adolescents Perinatal period Pregnant (6) Postpartum (4) Marital status of adolescents Married (5) Unmarried (5) Education level Primary (7) Secondary (3)	16–19	10:0
Family members	20–45	
Partners (3)		0:3
Parents (3)		3:0
Siblings (2)		2:0
Healthcare workers (10)	26–42	6:4

### Procedures

Written consent was obtained from adolescents ≥18 y of age. In Malawi, married participants <18 y of age are regarded as emancipated minors and can independently provide consent. Assent, backed by parental consent, was obtained from unmarried participants <18 y of age. We provided information sheets (in both English and Chichewa) that specified the study aims, benefits and potential risks to all participants (and parents of those who could not independently consent). All participants had an opportunity to ask questions before providing consent. During workshops (see Table 3), discussions were structured around the Template for Intervention Description and Replication (TIDieR) checklist.^22^

**Table 3.** Content of the co-design workshops

Workshop	Content
Workshop 1	The first round of ideation workshops was conducted to generate intervention ideas aimed at addressing the challenges that emerged during in-depth interviews. The initial structure of the intervention was informed by previous formative work as well as novel ideas generated by the stakeholders.
Workshop 2	The second round of ideation workshops was conducted to resolve contrasting views that emerged from the different stakeholder groups during the first round of the codesign workshops. A consensus on what to include was reached by listing all possible alternatives on flip charts, discussing pros and cons and selecting the ones that were more practical based on available resources. Participants contributed their ideas either by using sticky notes or by engaging in verbal discussions. Results from the first and second workshops were synthesized and an intervention ‘blueprint’ based on the TIDieR guidelines was developed.
Workshop 3	Samples of activity sheets were presented to participants during the third round of workshops to elicit user feedback for the refinement of the intervention ‘blueprint’. Some changes were suggested for the worksheets as follows: the worksheets should be visually appealing and must not look ‘too serious’ (e.g. by making them more colourful and easier on the eye) and the activities should be broken down into short manageable steps with concise instructions for each step.

The workshops were conducted face to face in a private space at the health facility. All workshops were audio recorded. Each workshop lasted approximately 2 h. All workshops were conducted in Chichewa (as per participants’ preference) and were facilitated by the first author (WM). Recordings were transcribed verbatim and subsequently translated into English by WM before proceeding with data analysis.

### Data analysis

The qualitative data from the workshops was synthesized using Framework Analysis,^23^ following the steps of transcription, familiarization, coding, developing and applying an analytical framework, charting and interpreting the data. The analysis followed a deductive approach where findings were organized around research questions and categorical labels from the TIDieR checklist that related to intervention characteristics. Data were presented in a narrative form in which we aimed to convey the key preferences of the stakeholders in relation to these different dimensions of intervention descriptors. We also provided a tabular summary of these descriptors in the form of a ‘blueprint’ (Table 4).

**Table 4.** ‘Blueprint’ of the FOR MAMA intervention


**Theoretical components** Stress-coping theory
**What?** Intervention components *Non-specific elements*: Active listening, empathy, eliciting commitment, normalization, discussing advantages of the intervention and barriers to engagement *Specific elements*: Emotion-focused coping (behavioural activation, relaxation techniques, anger management techniques) Problem-focused coping (problem-solving, financial literacy) Building social support/relationships (interpersonal skills; assertive communication skills) *Other in-session techniques*: Goal setting, psychoeducation, reviewing homework, giving praise to motivate, providing suggestions
**Who?** Delivery agent: Non-specialist health workers with at least 1 y of experience working with pregnant adolescents, but with no formal training related to mental health Selection criteria: Maternal health workers interested in acting as guides to volunteer Demographics: Bilingual (English and Chichewa speaking), >18 y of age, mixture of females and males Notable facilitators related to the choice of delivery agent: Experience working with pregnant adolescents, familiarity with the communities Notable barriers related to the choice of delivery agent: Existing workload at the health centre Mitigation: Intervention to be delivered on weekends, outside the normal work schedule
**Where?** Intervention setting: Both clinic and community based Rationale for the setting: Mitigate the distance barrier for pregnant adolescents Notable facilitators: Existing community structures where follow-up group and 1:1 sessions can be held Notable barriers related to the setting: Concerns about stigma from the community
**How?** Intervention characteristics *Delivery format*: Group, individual, face-to-face, telephone (on-demand) *Duration of intervention*: 5 weeks *Dosing:* Five follow-up sessions delivered weekly *Length of sessions*: 30–45 min for group sessions; 20–30 min for individual sessions *Materials*: Participants will receive four printed colour FOR MAMA booklets that give an overview of the program and explain the specific elements of the intervention using stories and corresponding practice activities; guides will receive a session-by-session manual, a progress monitoring tool and session record forms
**Methods for tailoring** Flexible rescheduling of follow-up sessions for those who miss the initial scheduled session Availability of on-demand telephone sessions for adolescents who miss the scheduled follow-up session or those who need assistance before the scheduled follow-up session

To improve reliability, the lead author (WM) and co-author (DG) independently read three transcripts (one from each stakeholder group) and manually coded the transcripts line by line. No coding software was used. The codes were compared and organized within a broad thematic framework reflecting intervention parameters set out in the TIDieR checklist. Once a consensus on the coding framework was reached, WM proceeded to assign codes to the rest of the transcripts, with feedback from the senior authors (DG and DM). To further enhance credibility and authenticity, findings were triangulated across three of the stakeholder groups and then discussed with the wider group of study co-authors.

## Results

### What intervention components might work best to address common mental problems among adolescents in Malawi?

Adolescents and service providers emphasized the need for information on causes, symptoms, prevention and coping strategies for common mental health problems. This was reiterated by service providers who stated that equipping adolescents with this information could promote help-seeking behaviour. Suggested coping strategies were problem-focused and emotion-focused.

During the stakeholder engagement, findings from the initial formative research^19^ on psychosocial risk and protective factors for perinatal common mental health problems were presented. Additionally, evidence-based intervention elements for perinatal interventions identified from the literature were shared. Stakeholders then generated and prioritized strategies to mitigate these risk factors and prevent common mental disorders among perinatal adolescents. Through a participatory process, participants contributed suggestions to address identified risk factors, engaged in verbal discussions to refine and prioritize ideas and voted to select the most urgent and useful strategies. Participants voted by placing sticky notes against their most preferred problem-focused and emotion-focused intervention elements. The elements were then ranked based on the number of votes they received.

#### Problem-focused strategies

Practical advice on how adolescents can deal with various challenges (problem solving) associated with pregnancy and childbirth was strongly recommended by all stakeholders. The need for financial literacy was also emphasized by all stakeholders since financial struggles were common among perinatal adolescents. Service providers emphasized the need for adolescents to develop interpersonal skills to expand their social networks and reduce social isolation.

#### Emotion-focused strategies

Service providers highlighted the need for behavioural activation to help adolescents who have lost interest in activities or use negative coping mechanisms. Skills to help adolescents manage different emotions (e.g. anger management skills) were suggested by adolescents and family members. The research team suggested relaxation techniques to address physiological symptoms, and this was endorsed by all stakeholder groups.

### Who are the potential non-specialists that could credibly deliver the intervention?

Most adolescents and family members indicated a strong preference for healthcare workers as intervention providers. Health surveillance assistants (HSAs) and community nurses who provide maternal health services were particularly preferred by adolescents. HSAs are primary health workers who mainly work in the communities to conduct health promotion activities, including monitoring mothers’ and children's health, providing vaccinations, promoting environmental health and tracing outbreaks of infectious diseases. Although some adolescents were open to having peers with experience of teenage pregnancy or older women as intervention providers, others stated that they would not be comfortable opening up to peers due to concerns about unwanted disclosure of private information outside of the helping relationship. Adolescents felt that HSAs and community nurses would be less likely to disclose sensitive information as they were aware of systems that hold HSAs and community nurses accountable or where they could report if a healthcare worker breached confidentiality. In addition, some adolescents and family members felt healthcare workers’ broader training and experience would afford them greater foundational knowledge about mental and physical health and their interplay. Furthermore, adolescents felt that they were more connected to HSAs and community nurses since they regularly visited local communities.

To further mitigate concerns about confidentiality, it was suggested that both intervention providers and participants should be made aware of the implications of breaching confidentiality prior to the intervention and reporting systems for the participants should be outlined and explained. This involves adolescents reporting the incident to the research team through the contacts indicated on the information sheet or consent form. The lead researcher would interview both parties to investigate these claims. Once it has been established that the provider has indeed breached confidentiality, they will be removed from the study and reported to the officer in charge of the health centre for a further disciplinary hearing.

Although healthcare workers were willing to deliver the intervention, they were concerned about intervention activities interfering with their usual assigned duties. The arrangement to have the intervention delivered on weekends was agreed upon by all stakeholders. It was also suggested that the health workers should receive adequate training and remuneration for their time.

### How can the intervention be efficiently delivered to perinatal adolescents in Malawi?

#### Group vs individual format

There were mixed views on individual versus group formats. Some participants from each of the stakeholder groups preferred the group format, as it was seen as a source of shared informational and emotional support among adolescents. It was also considered to be more efficient, particularly by healthcare workers. Adolescents’ main concern with groups was difficulty in observing confidentiality among participants. Adolescents and family members who preferred individual sessions felt this format would ensure that everyone is heard and assisted, unlike group settings where some participants may be more outspoken. The consensus was that the intervention would include both individual and group sessions, with group sessions being used for common and less sensitive issues. Hence for the FOR MAMA intervention, the group format will be utilized for the first session (onboarding) and the last session (looking ahead), while the individual format will be utilized for the follow-up sessions to allow the participants to discuss their unique problems with the guides.

#### Self-help vs guided support

Guided and self-help formats had mixed views from the participants. Some adolescents preferred guided formats (i.e. intervention fully delivered by healthcare workers), as they felt they may not be able to understand intervention material independently. However, they were concerned about missing important points and forgetting what they learned during the sessions. Other adolescents and some family members preferred self-help formats, as they felt adolescents could go through the material at their own pace. Nevertheless, the need for feedback and guidance for the activities was emphasized. Healthcare workers favoured a self-help format to lessen their workload. However, they agreed that a follow-up is needed to check if adolescents understand the intervention material and can complete the activities correctly.

#### Online vs in-person delivery

All stakeholder groups advocated in-person delivery, with supplementary materials provided in printed booklets rather than digital formats. It was highlighted that adolescents would not ordinarily possess personal internet-enabled devices or have other means of gaining online access. Telephone contact was suggested as a contingency in cases where an adolescent is in urgent need of assistance outside of a scheduled session or to make up for a cancelled or missed session.

It was suggested that printed booklets should use simple non-technical language and contextually appropriate colour pictures with a limited amount of text. They also suggested having stories to help them understand different elements of the intervention. However, comic strips were ruled out because most adolescents found them hard to follow. Most adolescents reported that they were used to formats that had pictures with text below the pictures, as this is similar to what is in their school textbooks.

The consensus among the stakeholders was to use a blended approach (guided self-help) where participants will have to read booklets and complete activities independently, with healthcare workers providing brief weekly follow-up sessions (lasting 20–30 min). Suggested roles for healthcare workers included reviewing the activities from the previous week and providing feedback, troubleshooting and introducing the next booklet to adolescents.

#### Tailoring

It was suggested that the scheduling for follow-up meetings should be flexible, as adolescents might encounter unforeseen circumstances (e.g. sickness and funerals) that may prevent them from engaging with booklets or attending scheduled follow-up sessions. In the case where a participant fails to engage with the booklet but shows up for a follow-up session, it was proposed that the guide should allocate time during the session for the adolescent to engage with the booklet before proceeding with the usual plan for the session.

#### Timing

All three stakeholder groups suggested that an antenatal intervention would be especially beneficial, as this is a critical period for incipient social challenges (e.g. stigma and school dropout) that affect mental health in the longer term. Moreover, participants commonly noted that the target group consists primarily of first-time mothers who are more likely to be anxious about pregnancy and childbirth.

A discussion on whether participants should be recruited based on the severity of symptoms was held. All stakeholder groups agreed that all pregnant adolescents should be eligible to participate regardless of whether they have elevated symptoms or not. This is because most pregnant adolescents face a lot of challenges that might put them at risk of having mental health problems at some point in their pregnancy. Healthcare workers and adolescents also argued that offering the intervention to all pregnant adolescents can reduce mental health stigma, as those who are taking part in the intervention will not be seen as ‘abnormal’.

#### Duration and dosing

All stakeholder groups felt that a brief antenatal intervention would be more acceptable and feasible to attend than a protracted, multisession program, especially given other demands on pregnant adolescents’ time such as school, work and physical health checks. It was therefore agreed that the intervention would be delivered within 4–6 weeks. To ensure that participants stay motivated, weekly sessions (as opposed to biweekly or monthly sessions) were preferred by all stakeholders.

### Where will the intervention be delivered to ensure practicability for both adolescents and intervention providers?

There were contrasting views on where the intervention should be delivered. Some adolescents and healthcare workers felt that delivering the intervention at the health centre would reduce the stigma associated with mental health problems, as most people would not question them about going to the health facility. Health centres also provide a more private environment with fewer interruptions compared with home. In contrast, some adolescent participants preferred the convenience of a home-based intervention, since it is common to live far from the health facilities and they may not have money for transportation. However, health workers raised concerns over house visits, as they may have to travel long distances from one household to another.

The consensus among the stakeholders was that the group sessions would be delivered at the health centre, while the follow-up sessions would be delivered in the community. To deal with the concerns about home visits, it was suggested that participants would be divided into clusters according to the proximity of their villages. Each cluster would have a central meeting place within the community, e.g. a school block or community health post, where follow-up one-on-one meetings would take place. To address the issue of stigma, it was agreed that the intervention would be universal, i.e. it would be delivered to any pregnant adolescent regardless of whether she had elevated symptoms or not.

## Discussion

This is the first participatory study aiming to co-design a mental health intervention for perinatal adolescents in Malawi. We conducted a series of co-design workshops with multiple stakeholders (adolescents, their family members and healthcare workers) to understand their intervention priorities and preferences. We found that adolescents preferred a multicomponent intervention delivered during the antenatal period. There was a strong preference for using self-help booklets with the guidance of healthcare workers. Participants agreed that the intervention would be delivered in both group and individual formats, with group formats taking place at the health centre and individual follow-up sessions taking place in the participants’ communities. Suggested practice elements included psychoeducation, problem-solving, financial literacy, interpersonal skills, behavioural activation, relaxation and anger management.

Psychoeducation, behavioural activation and relaxation are all common practice elements in low-intensity psychosocial mental health interventions for adolescents.^24^ Financial literacy is a less common component, although previous research highlighted the importance of practical skills to address psychosocial stressors in adolescents.^25^ The specific inclusion of content on financial literacy complements the problem-solving element by introducing potential ‘solutions’ to manage common financial challenges.

An antenatal intervention was preferred, as respondents felt that adolescents face many challenges during pregnancy, including stigma, school dropout and the anxiety of childbirth. In addition, participants felt equipping adolescents with coping skills during the antenatal period may help them cope better with the challenges that they encounter in both the antenatal and postpartum periods. This is in line with a recent systematic review that found that preventive interventions delivered during the antenatal period were more effective in alleviating symptoms of depression and anxiety as compared with postpartum interventions, as they improve psychosocial functioning and health-seeking behaviours throughout the perinatal period.^26^ In addition, providing mental health care during the antenatal period may prevent the onset of postpartum depression and anxiety, thereby leading to positive maternal and child health and development outcomes.^27^ It was also agreed that the intervention would be offered to all pregnant adolescents regardless of whether they have elevated symptoms of depression and anxiety. This is in line with research that suggests that providing maternal interventions universally can help overcome the fear of stigma, which is a major barrier to accessing maternal mental health care.^28^ Although FOR MAMA targets the antenatal period, some young women may benefit from having follow-up sessions in the postpartum period. Future research should explore the intervention needs of adolescents in the postpartum period in order to consider what type of support might complement an antenatal intervention like FOR MAMA.

Although there were mixed views regarding guided versus self-help formats, the consensus among the participants was to use a blended approach where they would engage independently with the intervention material while also receiving guidance and corrective feedback from healthcare workers. Similar findings were reported in another study^29^ where self-help formats were more acceptable when blended with some guidance. Furthermore, evidence from a systematic review and meta-analysis^30^ showed an advantage in effectiveness and engagement when blended formats are used instead of pure self-help. The use of digital formats was also discussed. All stakeholder groups agreed on face-to-face formats, as adolescents may not have access to mobile phones or internet-enabled devices. A recent scoping review of the development and use of mobile mental health in LMICs^31^ found that although mobile mental health interventions were generally acceptable among adolescents, delivery was hindered by factors such as limited internet access, data costs and low mobile phone ownership. However, considering the rapid adoption of mobile technology in LMICs, it may be appropriate to consider digital delivery formats in future iterations of the FOR MAMA program.

Group formatting was mostly preferred due to its efficiency and ability to provide social and emotional support. A pilot study on group cognitive behavioural therapy for perinatal anxiety disorders^32^ found that women in the perinatal period found group interventions useful, as they provided a platform for women to discuss their challenges with other mothers, thereby making them feel less alone. However, those who preferred individual sessions cited the need for confidentiality and the need for their unique needs to be addressed as reasons for the preference. Similar results were reported in a study on perceptions and treatment needs of perinatal depression in Malawi, where women had concerns about confidentiality and whether it was culturally appropriate to talk about personal or family issues in a group setting.^33^

Previous research has shown that privacy concerns are common among adolescents.^34^ In the current study, privacy concerns were raised in relation to home vs clinic delivery and group vs individual formats. Privacy concerns were also a reason why peer counsellors preferred healthcare workers. While most participants felt that a community-based intervention would be more convenient for the adolescents, as this would address the issue of travelling long distances to the health centre, some adolescents preferred an intervention delivered at the health centre. The main reasons for this preference were the lack of privacy at home and that taking part in the intervention at the health centre would reduce the mental health stigma that they may face in the community. Indeed, while home delivery may be important in addressing distance barriers to maternal mental health care access, caution must be exercised due to the lack of privacy or confidentiality in the home setting.^27^

A key strength of this study is the use of a participatory approach to guide the development of the intervention. Another strength is the triangulation of data from multiple stakeholders and findings from past research and those from the current study. However, the study was not without limitations. First, there may be a sampling bias related to the fact that we did not involve adolescents who were disengaged from primary health services. This may lead to an incomplete understanding of the mental health needs of all adolescents, especially those most vulnerable or marginalized. That being said, the most recent Malawi Demographic and Health Survey suggests a high uptake of antenatal services among pregnant adolescents in Malawi (94.4%).^4^

Although we utilized co-designing, the end users of the intervention were not involved in the design of the research, data collection or analysis of data. We also did not purposively recruit adolescents with lived experience of perinatal mental health problems. This was mainly due to time and financial constraints, as this was part of doctoral research with a minimal budget. However, the formative research^19^ leading up to this study included perinatal adolescents with elevated symptoms of mental health problems. Some of these participants also participated in this co-design study. Therefore, we anticipate that the voices of those with lived experience of mental health problems were represented by these participants. Nevertheless, future studies should prioritize the integral involvement of young people with lived experience in all stages of the study, allocating sufficient resources (in terms of both time and finances) to ensure genuine co-production and maximize the validity and impact of the research findings. Lastly, all adolescents in this study were literate. Hence their views regarding the use of booklets may be biased and may not reflect the views of the larger population. Moreover, 25% of adolescent girls ages 15–19 y in Malawi are illiterate.^35^ Therefore, it is possible that during implementation, some adolescents may be excluded from the study due to their inability to read and write. For future studies, we intend to adapt the intervention material to include those who cannot read and write using strategies such as visual aids, theatre strategies and involvement of family members.

To ensure sustainability, we plan to integrate the intervention into existing healthcare systems in Malawi, leveraging current infrastructure and resources. Moreover, we plan to increase capacity by training healthcare workers as well as lay persons (e.g. peers and mothers with lived experience of teen pregnancy) who can work within the healthcare setting. Although participants in this study indicated a strong preference for healthcare workers as intervention providers, other interventions for youth mental health (such as the Youth Friendship Bench in Zimbabwe)^36^ have successfully engaged other cadres such as young lay health workers as intervention providers. Therefore, future FOR MAMA studies will explore the possibility of engaging other cadres of intervention providers as a strategy for scaling up, considering the overloading of the healthcare system in Malawi.

### Conclusions

The current study led to the development of a ‘blueprint’ for a multicomponent, guided mental health intervention for pregnant adolescents in Malawi. The findings are consistent with common elements in adolescent-focused interventions for perinatal mental health interventions globally.^15^ The next steps involve evaluating the feasibility, acceptability and potential impacts of the intervention.^37^ Furthermore, there is also a need to conduct a cost-effectiveness evaluation (including an economic analysis), as this is crucial for assessing the scalability of the intervention.

## Data Availability

Due to conditions of participant consent, the data from this study are not publicly available.
